# Variables that influence teachers’ practice of differentiated instruction in Chinese classrooms: A study from teachers’ perspectives

**DOI:** 10.3389/fpsyg.2023.1124259

**Published:** 2023-03-06

**Authors:** Meijie Bi, Katrien Struyven, Chang Zhu

**Affiliations:** ^1^Department of Educational Sciences, Vrije Universiteit Brussel, Brussels, Belgium; ^2^School for Educational Studies, Hasselt University, Hasselt, Belgium

**Keywords:** differentiated instruction, teachers’ practice, psychometric properties, DI-Quest instrument, Chinese secondary schools

## Abstract

As the diversity of students increases, differentiated instruction (DI) serves an increasingly significant function in meeting their individual learning needs. Emerging research has highlighted the value of inclusive teaching approaches to address students’ differences, such as DI. Therefore, it is important to quantify teachers’ DI thoughts and behaviors in classroom teaching. This study follows the original Differentiated Instruction Questionnaire (DI-Quest) to investigate the factors that influence teachers’ practice of DI, taking into account their teaching experience, class sizes and school locations. The sample comprised 1,935 teachers from 150 national lower secondary schools in six provinces of central and western mainland China. Exploratory factor analysis (EFA), confirmatory factor analysis (CFA), scale reliability, and invariance testing were conducted to explore and verify the factor structure of a Chinese mainland version of the DI-Quest (CN-DI-Quest). The empirical findings indicate that CN-DI-Quest is a valid and reliable instrument for future study of teachers’ DI philosophies, principles, and practice. Moreover, the results of structural equation modeling revealed that teachers’ practice of DI (i.e., adaptive teaching) was explained largely by their ethical compass, flexible grouping, output = input, teaching experiences, and class size. Notably, teachers’ practice of DI (i.e., adaptive teaching) could not be predicted by growth mindset and school location. This study addresses gaps in the literature, since it provides empirical evidence regarding DI in Chinese mainland schools, offers material and suggestions for future research, and provides recommendations useful to the professional advancement of Chinese teachers, including training programs and professional support.

## 1. Introduction

Student diversity within classrooms is rising both consistently and globally and provides challenges with far-reaching implications. Educators must implement appropriate teaching strategies to bridge student differences and ensure that all students are given maximum opportunity to learn (Unesco, 2017). Differentiated instruction (DI), which has emerged as an effective classroom practice for responding to individual differences and meeting students’ diverse learning needs, requires instructors to consider the differences among their students and to tailor their teaching practice in light of these (Adebayo and Shumba, 2014; Dixon et al., 2014). This study investigates DI and in doing so, uses the definition proposed by Tomlinson (2014, 2017), who describes DI as both a teaching philosophy and classroom practice, in which teachers are responsive, proactive, and positive in their accommodation and leverage of students’ differences.

Ultimately, the successful practice of DI depends on teachers, and it is therefore necessary to understand the underlying variables that influence their DI practice. More specifically, many researchers have reported that teachers’ DI beliefs, teaching experience, and class size are strongly associated with their DI practice (e.g., Suprayogi et al., 2017; Whitley et al., 2019). However, such research in mainland China has rarely been conducted. Furthermore, research investigating the relationship between school location and DI practice remains rare. The current study aims to explore the impact of teachers’ belief, teaching experience, class size, and school location on their practice of DI, and thus to provide new empirical evidence from the Chinese context and valuable suggestions for teaching practice, training, and future research.

## 2. Background and literature review

The increasing diversification of classrooms is a global phenomenon (Smale-Jacobse et al., 2019). However, the resulting challenges are perhaps more acute in China than elsewhere because, additionally, the Chinese government has introduced a policy named Learning in Regular Classrooms (LRC), which mandates the teaching of students with special educational needs in mainstream classrooms (Cheng, 2005; Shi and Hua, 2007; Deng and Harris, 2008). Thus, Chinese teachers must address and cater for a wide range of differences among learners, and ensure that all students have the best possible opportunities to learn (Yan and Hua, 2020). Clearly, DI is applicable here, and fortunately, the broad concept of DI is not new for Chinese teachers. A traditional Confucian belief, that education should be delivered in accordance with students’ individual characteristics and learning needs, has given the country a valuable platform upon which to build a modern iteration of DI (Lam et al., 2002). However, the development of the Confucian concept of education did not occur systematically, and until recently, it was seen as a somewhat abstract teaching principle. Furthermore, while modern DI has been empirically tested and frequently implemented in the West (Tomlinson, 1999, 2014), until now, most assessment of DI in Chinese societies has been conducted in Hong Kong (e.g., Yuen et al., 2022) and Taiwan (e.g., Hung and Chao, 2021), with little evidence gleaned from the Chinese mainland. Hence, this study hopes to provide a new viewpoint and understanding of teachers’ practice of DI on the Chinese mainland, and to explore the factors that influence teachers’ DI practice through connection of the original DI-Quest variables with teaching experience, class size, and school location (for which more detail is given in section 2.2).

### 2.1. The differentiated instruction-quest model

Since teachers play a significant role in DI, instruments have been developed to measure teachers’ DI practice, including the Differentiated Instruction Scale (Roy et al., 2013), DI practice (Letzel, 2019), and the DI-Quest instrument (Coubergs et al., 2017). Since the DI-Quest was developed to describe the extent to which teachers’ thought and performance by emphasizing their DI philosophies and principles, and was validated in Belgian (Coubergs et al., 2017) and Hong Kong (Yuen et al., 2022) schools, it was chosen for use in this study, as the main instrument to explore Chinese teachers’ DI philosophies and practices.

The DI-Quest model (Table 1) comprises five constructs: growth mindset, ethical compass, flexible grouping, output = input, and adaptive teaching (i.e., adaptive to students’ differences in readiness, interests, and learning profiles, which are, in turn, identified as three factors in the model; Coubergs et al., 2017). The first two constructs (i.e., growth mindset and ethical compass) are categorized as teachers’ philosophies of DI; the subsequent two (i.e., flexible grouping and output = input) are categorized as teachers’ principles on how to organize DI teaching; the last construct (i.e., adaptive teaching) is the practice of DI whereby teachers differentiate their practice according to students’ interests, readiness, and learning profiles (Coubergs et al., 2017). The last factor is considered crucial, since it acts as the “core function” of DI, implying that the core concept of the DI framework includes students’ learning differences in terms of interests, readiness, and learning profiles (Tomlinson and Moon, 2014; Tomlinson, 2017). Thus, the other four factors may be utilized to predict the last factor (Coubergs et al., 2017).

**Table 1:** The description of DI-Quest instrument.

	**Variables**	**Number of items**	**Example item**
**Philosophies of DI**	Growth mindset	5	A teacher’s belief in the competences of a student can influence their intellectual capacities
	Ethical compass	6	The curriculum does not provide any flexibility to cope with an individual student
**Principles of DI**	Flexible grouping	8	Working in heterogeneous groups gives students the opportunity to learn from each other
	Output = input	4	I use assessment to gain insight into the learning processes of my students
**The practice of DI**	Adaptive teaching	8	Knowing my students, I select the learning content, materials, and teaching methods

#### 2.1.1. Philosophies of differentiated instruction: Growth mindset and ethical compass

According to the definition provided by Dweck (2006), growth mindset is an implicit belief concerning the stability of capability. Teachers with a growth mindset generally embrace students’ interests, readiness, and learning profiles as the basis for differentiating their teaching, which may lead students to achieve at higher levels than they have done, or would do, otherwise (Hattie, 2005; Coubergs et al., 2017). In contrast, teachers who maintain a fixed mindset are more likely to believe that students’ learning success is determined by their attributes, such as talents and intelligence. Those with fixed mindsets may consider intellectual capability as static, and attach little, if any, significance to the skills and effort applied by their students (Lynott and Woolfolk, 1994). Consequently, in the classroom environment, they use controlling teaching practices, rather than devise a competitive learning environment (Lambert, 1999).

The term ethical compass refers to whether teachers consider the (a) curriculum or (b) their observation of the students’ learning as a compass for teaching (Tomlinson and Imbeau, 2010). It reflects how flexibly a teacher adapts the curriculum and makes adjustments that meet students’ learning needs (Coubergs et al., 2017). Teachers who restrict their approach to strict pursuit of the curriculum, without regard to learners’ needs, may assume that student performance depends on external factors, such as government policies and regulation, structure, or discipline (Coubergs et al., 2017). Therefore, teachers with curriculum-centered beliefs are less inclined to differentiate their classroom teaching. However, teachers who tailor their instructions more precisely, writing lesson plans and designing exploratory activities to expedite students’ learning goals, are said to hold student-centered beliefs (Tomlinson and Imbeau, 2010).

#### 2.1.2. Principles of differentiated instruction: Flexible grouping and output = input

Teachers who are proficient in DI practice should plan student study groups with a flexible approach (Tomlinson, 2001); flexible grouping refers to a practice of grouping students homogeneously and heterogeneously according to the learners and targets involved, and of switching these groups flexibly in classrooms so that students experience both independent learning and work in various cooperative groups (Tomlinson et al., 2003). Flexible grouping lets teachers monitor and evaluate students in various learning contexts, *via* provision of diverse learning materials and target tasks (Tomlinson, 2001; Whitburn, 2001). Research indicates that flexible grouping of students tends to work best when used with appropriately differentiated materials, profiles, methods, activities, and learning goals (Tieso, 2005; Aliakbari and Haghighi, 2014).

Output = input refers to the principle that teachers should plan their teaching (input) according to students’ classroom performance (output), which helps teachers to understand their students’ learning progress and assist them accordingly (Hall, 2002). Successful practice of DI requires teachers to provide students with ongoing feedback on their performance, both during and after classroom activities (Hattie, 2012). This also helps teachers to prepare appropriately for subsequent lessons (Hattie, 2009). The notion that the application of adaptive teaching should be based on the learning differences of learners and appropriate feedback is confirmed in the work of Coubergs et al. (2017).

#### 2.1.3. Practice of differentiated instruction: Adaptive teaching to accommodate learning differences

The term adaptive teaching refers to the proactive and positive actions that teachers take in response to students’ learning needs (Parsons, 2008). According to Tomlinson et al. (2003), teachers can do helpfully adapt their teaching practices in light of three specific types of learning need, namely, readiness, interests, and learning profiles. Thus, to accommodate differences in students’ readiness to study, their previous knowledge could be linked to their learning goals in fields of study, subject areas, and topics based on their current learning status (Woolfolk, 2010). By taking differences in readiness into account, teachers can provide greater possibilities for every learner to achieve the present and desired levels of learning. In addition, the need to respond to students’ interests suggests teachers should offer topics, contents, or activities that interest students, since this is associated with positive learning experiences, greater levels of student engagement, and productivity (Eisenberger and Shanock, 2003; Woolfolk, 2010). Finally, the differences exhibited by students in terms of learning profiles indicate diverse approaches or modes of learning that consider the combined outcomes of several factors; for instance, contexts, topics, gender, and intelligence (Tomlinson et al., 2003). Teaching that caters for the differences between students’ learning profiles is likely to improve their outcomes considerably (Perry et al., 2004).

### 2.2. Variables influencing differentiated instruction practice

The literature indicates that the practice of DI and accommodation of differences in readiness, interests, and learning profiles can exert a positive influence on students’ academic achievement, classroom engagement, learning interests, enthusiasm, and self-confidence (e.g., Tulbure, 2011; Bal, 2016; Eysink et al., 2017). However, this process is not necessarily straightforward, and research has reported a set of complex variables, at both teacher-levels and context-levels, that influence teachers’ practice of DI.

Teachers’ belief in DI greatly affects their DI practice in classrooms. Studies have reported that the instructional outcomes of teachers, such as their actions and decisions in the classroom, are guided by their educational ideas, thoughts, and opinions (He and Levin, 2008; Cross, 2009). Whitley et al. (2019) conducted mixed methods research within K-12 settings and found a significant correlation between teachers’ DI beliefs and their DI practices. Suprayogi et al. (2017) found similar in a survey of 604 teachers, stating that teachers’ differing beliefs in constructivist ideas and self-efficacy hinder the practice of DI. Furthermore, a case study of eight teachers revealed that teachers’ classroom practice is enhanced by their positive perceptions of DI (Sibanda, 2021), and that teaching experience is a factor affecting DI practice (Casey, 2011; Dixon et al., 2014). Teachers with at least 5 years’ experience applied DI more often than their less experienced peers (Davis, 2013). Corresponding outcomes were reported by Sheehan (2011), who found that teachers with at least 8 years’ experience maintained a positive attitude toward DI practice, while Burkett (2013) also linked teaching experience to the use of DI strategies. Generally speaking, the literature has suggested that experienced teachers use a more extensive range of educational practices, which helps them to optimize their DI instructions and strategies (Liu et al., 2010; Ginja and Chen, 2020). However, some scholars have claimed that teaching experience has no significant correlation with DI practice (e.g., Donnell and Gettinger, 2015; Merawi, 2018).

Moreover, the practice of DI is intricately associated with classroom size and school location. Large class size has been identified as a barrier to implementing DI (e.g., Wan, 2016; De Jager, 2017). Often, as class size increases so does diversity in students and learning needs (Dixon et al., 2014). This makes class management more complicated for teachers, who accordingly refrain from using DI (Aldossari, 2018; Moosa and Shareefa, 2019). Regarding school location, few studies have examined the correlations among DI practice, student performance, and teacher-related parameters in rural or urban schools (D'Angelo, 2006; Wu, 2017; Goddard and Kim, 2018; Goddard et al., 2019). Until recently, it was unknown whether teachers’ DI practice varied between rural and urban schools, or whether school location is an influential variable in teachers’ DI. A recent study has explored factors affecting DI practice in the rural and urban schools but found no significant difference between them (Lavania and Nor, 2021).

## 3. Materials and methods

### 3.1. Research questions

**Figure 1:**
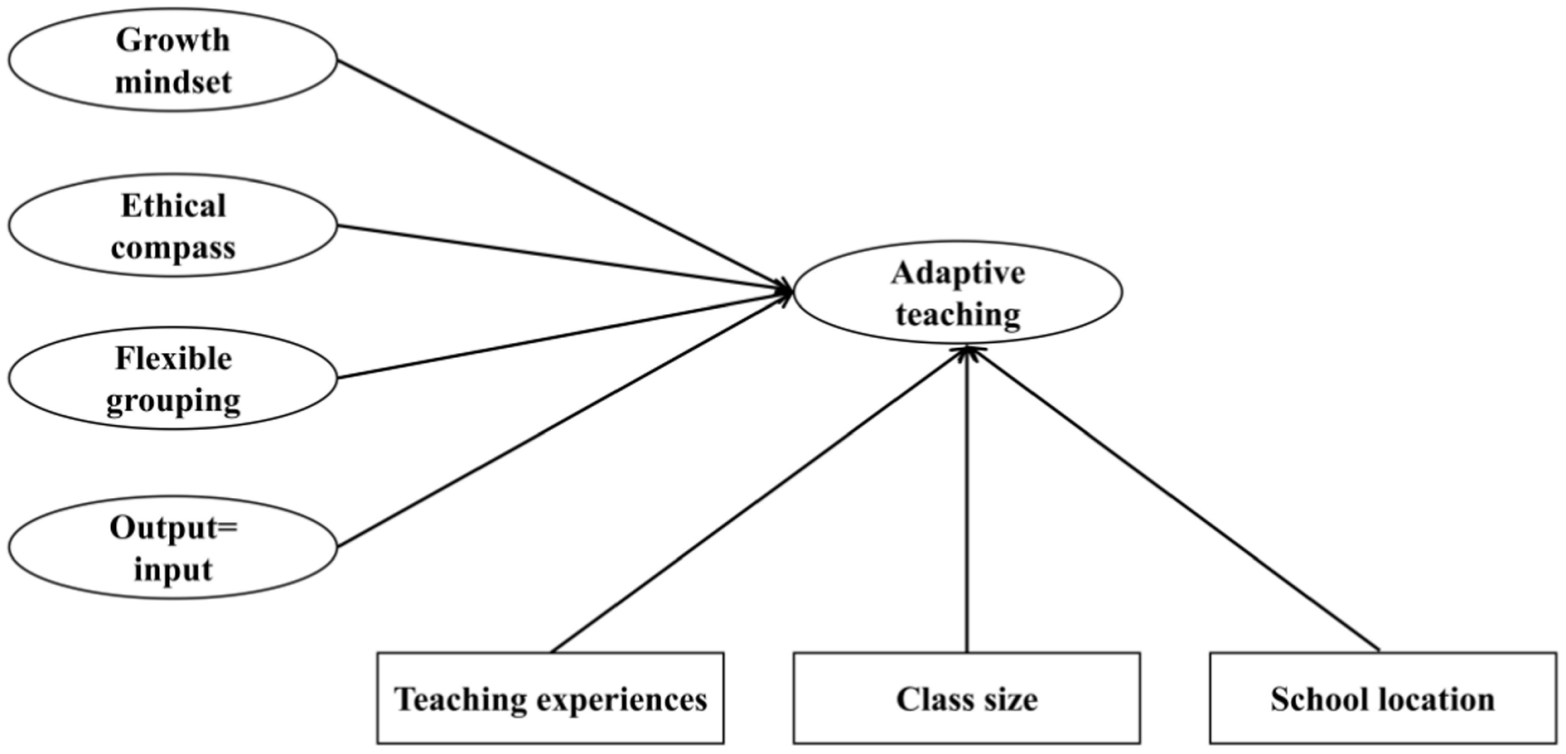
The conceptual research model of this study.

Following the DI-Quest model, this study aims to examine the effect of teachers’ self-reported DI philosophies and principles on their DI practice, while considering their teaching experiences, class size, and school location (see Figure 1). Two research questions are posed:

1. Are the five-factor structures of the original DI-Quest instrument fit for Chinese mainland schoolteachers?
2. How do teachers’ philosophies, principles, teaching experience, class sizes, and school locations relate to their self-reported DI practice?

### 3.2. Research context and procedure

For this large-scale study, we selected six provinces in central and western China as the research area. All districts in the research area have been supported by government programs to encourage the use of DI, and participating schools were selected randomly, without considering the student number, class size, and geographical locations within the selected provinces. The study was conducted in national lower secondary schools (OECD, 2021), which provide the final 3 years of compulsory education for Chinese students, culminating in the national upper secondary school selection examination.

The study ran from September 2019 to December 2019. The researcher presented the research proposal to the school authorities and requested their participation through emails and/or calls. Some declined, questioning the inclusion of political elements in the questionnaire. However, once the expression of two items (items 9 and 11) had been modified and permission secured from the principals, an online hyperlink was emailed to school principals, who then invited teachers from Grades 7–9 to complete the survey voluntarily, in their spare time.

### 3.3. Sample size and demographics

We used the online Raosoft sample size calculation methodology to determine the sample size; it suggested a minimum of 1,676 participants (margin error alpha = 0.03, the confidence level is = 99%, total population = 20,000, the response of distribution = 65%; Raosoft, 2004). Therefore, 1,935 teachers were invited to participate, from 150 schools in six provinces throughout central and western China. Approximately 1,694 teachers from national lower secondary schools provided the information for every variable. After deletion of unusable data, 1,689 responses were used for further analysis.

Of the valid responses, 1,040 were from female teachers and 649 from male teachers. Most teachers (1,479) had a bachelor’s degree while the number of teachers holding under bachelor and master’s degrees was small. 758 teachers had over 20 years of teaching experience. Respondents’ class sizes ranged from 15 to 79 students: a class with more than 55 students is considered a large class—in China, the average class size is 45 for elementary schools and 50 for lower secondary schools (Jiacheng and Jing, 2013). Respondents having class sizes of 15–40 and 41–55 were 24.6 and 59.6%, respectively. Large classes (>56) accounted for 15.7%. Regarding geographical distribution, 29% of the sample worked at rural schools; teachers working in town and city schools constituted 47.4 and 23.6%, respectively (Table 2).

**Table 2:** Demographic characteristics of the samples.

**Demographics**		**Sample size**	**Proportion (%)**
**Gender**			
	Male	649	38.4
	Female	1,040	61.6
**Age**
	≤35	556	32.9
	36–50	802	47.5
	≥51	331	19.6
**Academic qualification**			
	Under Bachelor’s degree	67	4.0
	Bachelor’s degree	1,479	87.6
	Master’s degree	143	8.5
**Years of teaching experiences**			
	≤5	286	16.9
	6–20	645	38.2
	≥20	758	44.9
**Class size**			
	15–40	416	24.6
	41–55	1,007	59.6
	≥56	266	15.7
**School location**			
	Rural	490	29.0
	Town	800	47.4
	City	399	23.6

### 3.4. Research instrument

The online questionnaire survey comprised two parts: (i) basic demographic information and (ii) the DI-Quest instrument. Data on the background variables of teachers and schools, including gender, age, academic qualification, years of teaching experience, class size, and school location, were collected. In the event, that the respondents taught more than two classes, the variable (class size) chosen was the class with the largest number of students. The second part of the questionnaire comprised the original version of the DI-Quest instrument (Coubergs et al., 2017), whereby teachers report the extent to which they differentiate their practices in classrooms according to the philosophies and principles of DI. The instrument was launched in Belgium (Coubergs et al., 2017), then validated in Hong Kong (Yuen et al., 2022). It comprises five dimensions with 31 items. An ordinal frequency rating scale (1 = totally disagree, 5 = totally agree; 1 = never, 5 = always) is used to measure growth mindset, ethical compass, and flexible grouping, as well as output = input and adaptive teaching. Notably, since the two items of growth mindset and six items of ethical compass express the reverse meaning, these items were reversed for further analyses. Table 1 shows the number of items and example items for each dimension.

#### 3.4.1. Translation of the differentiated instruction-quest instrument

We used forward-backward translation procedures to translate the original DI-Quest instrument (Behling and Law, 2000). The first author translated the initial version into Chinese, then invited two professors in educational fields and one expert in Linguistics to proofread it. Following discussion, minor changes in wording and expression were made to clarify the meaning and linkage with the (Chinese national lower secondary school) context. The Chinese version of the DI-Quest instrument was then given to another two Chinese experts in education, who translated it back into English. The first author and two professors worked together to compare and check these two translations, and differences between versions were discussed until an agreement was reached.

### 3.5. Data analysis

This study firstly assessed whether the univariate and multivariate normality of the data obtained met the general requirements. Regarding univariate normality, no standardized skewness (range − 0.935 to 0.180) and kurtosis (range − 0.851 to 1.885) values for each item fell outside the range of −3 to +3, indicating that no violation of univariate normality existed (Kim, 2013). Regarding multivariate normality, the Mardia’s multivariate kurtosis (MK) test showed a significant result (MK = 202.925; z-statistic = 91.64, *p* < 0.001). The expected value of multivariate kurtosis can be calculated through a formula, p (p + 2), in which p refers to the number of observed variables (Cain et al., 2017). After comparing the observed value and expected MK, we found deviation from multivariate normality in this study. We used maximum likelihood estimation (MLR) to deal with the deviation in the subsequent confirmatory factor analysis (CFA), since it produced robust standard errors and rescaled test statistics (Curran et al., 1996).

Concerning the first research question, to examine the psychometric properties of the DI-Quest instrument in the Chinese mainland school context, one-half of the data set (*n* = 845) was randomly selected for the exploratory factor analysis (EFA) in SPSS 26, to identify the factor structure of the 31 items. The rest of the data set (*n* = 844) was evaluated with CFA in Mplus 8.7, to confirm the factor structure through EFA. Before employing EFA, the Kaiser-Meyer-Olkin (KMO) and Bartlett’s test of sphericity were applied, to demonstrate sampling adequacy (Pallant, 2020). For the EFA, principal component analysis (PCA) with an oblique rotation method was used to verify the structure of the 31 items (Fabrigar and Wegener, 2012). Factors with eigenvalues greater than 1 (Kaiser, 1960) and factor loadings above 0.4 were retained (Tabachnick et al., 2013). Furthermore, CFA was used to identify the factor solution from EFA, and criteria for evaluating the model fit were: Satorra-Bentler Scaled Chi-Square to degrees of freedom ratios (SBχ^2^/dƒ) <0.3, a root mean square error of approximation (RMSEA) <0.08, comparative fit index (CFI) >0.9, Tucker-Lewis index (TLI) >0.9, and standardized root mean square residual (SRMR) <0.08 (Hu and Bentler, 1999). In addition to EFA and CFA, scale reliability was measured through computation of the Coefficient H, since the estimate of Cronbach’s alpha of checking the internal reliability of the factor structures usually generates the lowest possible value (Sijtsma, 2009).

We also conducted invariance testing of the factor structure across gender, age, and school location, using multi-group CFA. The steps of configural, metric, and scalar were included in measurement invariance tests. The differences in CFI (ΔCFI) and RMSEA (ΔRMSEA) were calculated for examination of differences in model fit. If the value of (ΔCFI) is ≤0.01, and the value of RMSEA is <0.05, the hypothesis of invariance should be accepted (Cheung and Rensvold, 2002). Subsequently, descriptive statistics and a correlation matrix for CN-DI-Quest were reported.

For the second research question, we used a structural equation modeling in Mplus to explore the impacts of teachers’ philosophies (i.e., growth mindset and ethical compass), principles (i.e., flexible groping and output = input), teaching experience, class size, and school location upon their reported DI practice (i.e., adaptive teaching).

## 4. Results

### 4.1. Exploratory factor analysis, confirmatory factor analysis, scale reliability, and invariance testing

**Figure 2:**
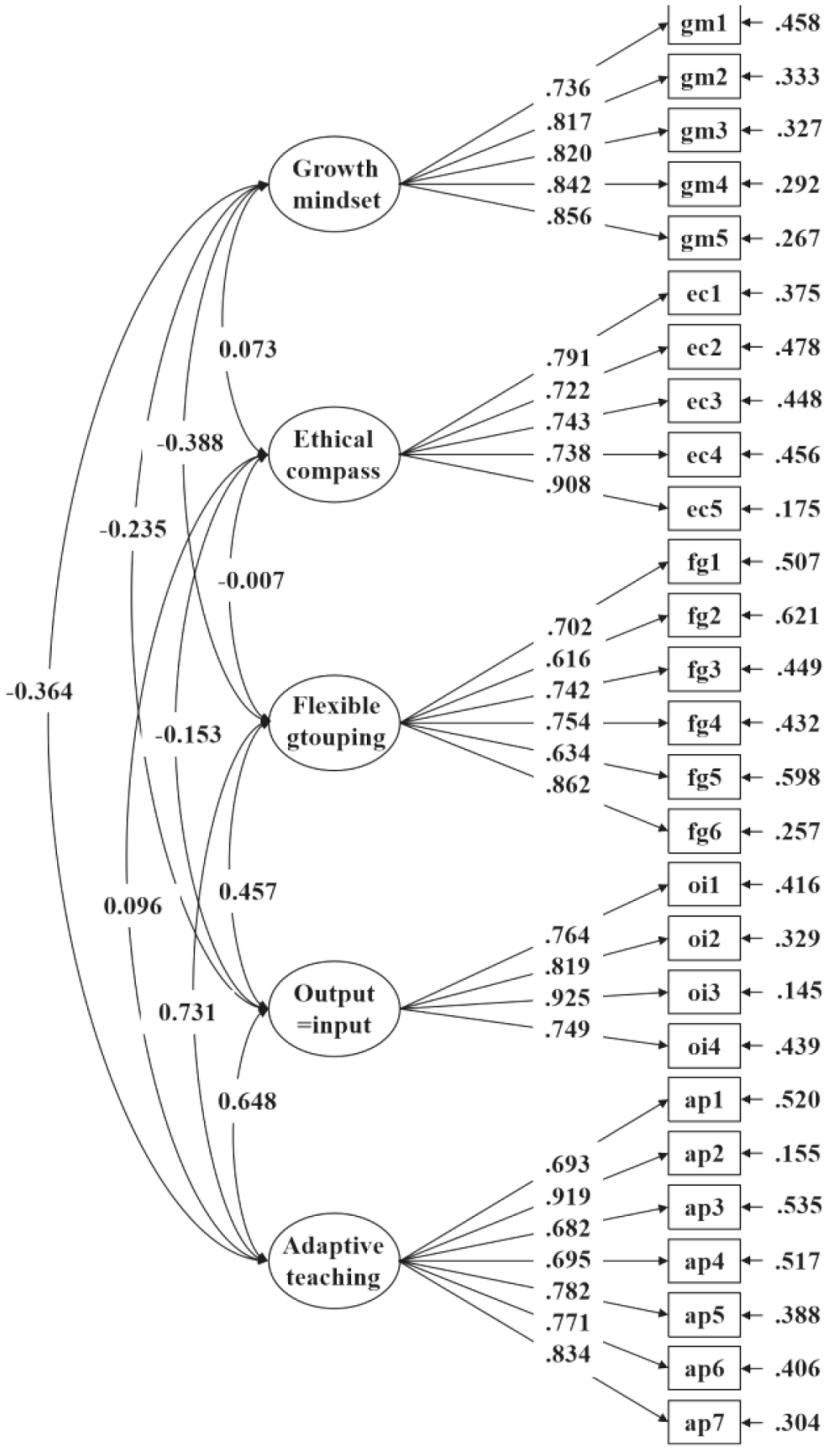
The measurement model of Chinese version DI-Quest instrument; *N* = 844. Coefficients presented are standardized estimates.

Regarding data suitability, the value of KMO measurement and Bartlett’s test of sphericity gave significant results (KMO = 0.920, χ^2^ (465) = 14719.493, df = 465, *p* ≤ 0.001; Pallant, 2020). A five-factor solution was yielded through the initial eigenvalue analysis based on the aforementioned factor and item retention criteria. These five factors accounted for 16.59, 14.01, 13.36%, 13.38, and 11.01% of the variance, respectively (Table 3). Four items from the original DI-Quest questionnaire were eliminated (EC2, FG4, FG8, and AP4), because their factor loadings had values lower than 0.4 (Tabachnick et al., 2013). The 27 retained items were re-ordered and the standardized factor loading values can be found in Table 4. Based on the five constructs with 27 items identified in EFA, we conducted CFA to further validate them (Figure 2). No further items were deleted, and sufficient fit was derived from the CFA modeling (SBχ^2^ = 675.823, df = 314, CFI = 0.967; TLI = 0.963; RMSEA = 0.037; SRMR = 0.033; Table 5; Hu and Bentler, 1999).

**Table 3:** The factor loadings in EFA for the initial and final round.

**Factor**	**Item**	**Initial: Factor loading**					**Final (remove items): Factor loading**					**% of variance explained**
		**1**	**2**	**3**	**4**	**5**	**1**	**2**	**3**	**4**	**5**	
**Adaptive teaching**	AP1	**0.720**	0.189	0.026	−0.029	0.206	**0.717**	0.182	−0.025	0.024	0.211	16.588%
	AP2	**0.836**	0.315	0.134	−0.024	0.216	**0.846**	0.301	−0.017	0.134	0.204	
	AP3	**0.650**	0.222	0.094	−0.095	0.186	**0.656**	0.208	−0.090	0.096	0.182	
	AP4	0.084	0.122	0.062	0.048	0.398	–	–	–	–	–	
	AP5	**0.619**	0.258	0.081	−0.032	0.246	**0.632**	0.250	−0.024	0.080	0.227	
	AP6	**0.759**	0.209	0.115	−0.012	0.159	**0.769**	0.188	−0.008	0.113	0.147	
	AP7	**0.726**	0.283	0.133	−0.085	0.184	**0.740**	0.269	−0.077	0.134	0.165	
	AP8	**0.715**	0.347	0.058	−0.051	0.234	**0.731**	0.335	−0.044	0.059	0.211	
**Flexible grouping**	FG1	0.227	**0.730**	0.181	−0.051	0.066	0.253	**0.728**	−0.042	0.185	0.020	14.014%
	FG2	0.301	**0.673**	0.101	0.017	0.124	0.321	**0.672**	0.025	0.104	0.089	
	FG3	0.269	**0.744**	0.118	0.061	0.115	0.300	**0.740**	0.072	0.126	0.061	
	FG4	0.005	0.097	0.058	0.077	0.282	-	-	-	-	-	
	FG5	0.211	**0.705**	0.150	0.090	0.196	0.238	**0.710**	0.103	0.154	0.147	
	FG6	0.170	**0.611**	0.161	−0.011	0.191	0.183	**0.618**	0.001	0.172	0.167	
	FG7	0.217	**0.832**	0.100	0.048	0.207	0.247	**0.836**	0.062	0.108	0.151	
	FG8	0.193	0.321	0.026	0.055	−0.023	-	-	-	-	-	
**Growth mindset**	GM1	0.185	0.027	**0.738**	0.030	0.004	0.193	−0.002	0.043	**0.745**	0.002	13.357%
	GM2	0.075	0.173	**0.809**	−0.024	0.078	0.074	0.167	−0.007	**0.817**	0.091	
	GM3	0.041	0.193	**0.842**	−0.014	0.072	0.056	0.182	0.004	**0.844**	0.058	
	GM4	0.054	0.235	**0.810**	−0.030	0.120	0.064	0.229	−0.012	**0.814**	0.111	
	GM5	0.070	0.179	**0.876**	0.000	0.048	0.081	0.160	0.020	**0.889**	0.043	
**Ethical compass**	EC1	−0.037	0.056	0.016	**0.857**	0.080	−0.032	0.055	**0.860**	0.001	0.051	13.384%
	EC2	0.044	−0.025	0.384	0.104	0.127	–	–	–	–	–	
	EC3	−0.030	0.071	−0.009	**0.813**	0.025	−0.024	0.062	**0.815**	−0.015	0.003	
	EC4	−0.076	−0.038	0.027	**0.812**	0.080	−0.076	−0.036	**0.815**	0.014	0.063	
	EC5	−0.047	0.061	0.031	**0.802**	0.105	−0.040	0.063	**0.807**	0.019	0.076	
	EC6	−0.019	0.011	0.049	**0.910**	0.078	−0.018	0.008	**0.911**	0.031	0.058	
**Output = input**	OI1	0.288	0.101	0.108	0.022	**0.755**	0.282	0.136	0.044	0.108	**0.761**	11.016%
	OI2	0.263	0.061	0.078	0.071	**0.817**	0.255	0.106	0.094	0.074	**0.822**	
	OI3	0.280	0.073	0.067	0.056	**0.851**	0.266	0.120	0.077	0.060	**0.865**	
	OI4	0.255	0.122	0.056	0.057	**0.756**	0.245	0.164	0.078	0.053	**0.763**	

*N* = 845. Factor loadings above 0.4 are bolded.

**Table 4:** Standardized factor loading and scale reliability.

**Factor**	**Item**	**Factor loading**	**Coefficient H**
**Growth mindset**	GM1	0.736	0.912
	GM2	0.817	
	GM3	0.820	
	GM4	0.842	
	GM5	0.856	
**Ethical compass**	EC1	0.791	0.908
	EC2	0.722	
	EC3	0.743	
	EC4	0.738	
	EC5	0.908	
**Flexible grouping**	FG1	0.702	0.885
	FG2	0.616	
	FG3	0.742	
	FG4	0.754	
	FG5	0.634	
	FG6	0.862	
**Output = input**	OI1	0.764	0.914
	OI2	0.819	
	OI3	0.925	
	OI4	0.749	
**Adaptive teaching**	AP1	0.693	0.931
	AP2	0.919	
	AP3	0.682	
	AP4	0.695	
	AP5	0.782	
	AP6	0.771	
	AP7	0.834	

*N* = 844. The value of standardized factor loading of each item is significant.

**Table 5:** Fit indices for the model 1 and model 2.

**Index**	**χ**^**2**^_**S-B**_	**df**	**χ**^**2**^_**S-B**_**/df**	**CFI**	**TLI**	**RMSEA [90%CI]**	**SRMR**	**BIC**
**Model 1**	675.823	314	2.152	0.967	0.963	0.037 [0.033,0.041]	0.033	44760.395
**Model 2**	1132.465	412	2.749	0.970	0.966	0.032 [0.030,0.034]	0.050	89813.435

Model 1: Results from EFA, five-factor structure with 27 items, CFA sample (*N* = 845).

Model 2: Results from five-factor structures of Chinese DI-Quest and three background variables: teaching years, class size and school location, SEM sample (*N* = 1,689).

We also tested scale reliability, and the score of Coefficient H ranged from 0.89 to 0.93, indicating that the Chinese version of the DI-Quest instrument is highly reliable (Table 5). Regarding invariance testing, as mentioned earlier, strong invariance was achieved for three groups (i.e., gender, age, and school location) after comparing the results of configural, metric, and scalar steps in this study (Table 6).

**Table 6:** Results of invariance analysis.

**Model**	**χ**^**2**^_**SB**_	**df**	***p***	**CFI**	**△CFI**	**RMSEA [90%CI]**	**△RMSEA**	**SRMR**	**BIC**
**Gender**									
Configural	1304.437	628	<0.001	0.969		0.036 [0.033,0.038]		0.034	93255.107
Metric	1326.911	650	<0.001	0.969	0.000	0.035 [0.032,0.038]	−0.001	0.036	93122.468
Scalar	1369.504	672	<0.001	0.968	−0.001	0.035 [0.032,0.048]	0.000	0.036	93001.448
**Age**									
Configural	1609.899	942	<0.001	0.970		0.035 [0.033,0.038]		0.038	93880.820
Metric	1651.434	986	<0.001	0.970	0.000	0.035 [0.032,0.038]	0.000	0.041	93610.096
Scalar	1691.617	1,030	<0.001	0.970	0.000	0.034 [0.031,0.037]	−0.001	0.042	93319.329
**School location**									
Configural	1726.765	942	<0.001	0.965		0.038 [0.036,0.041]		0.040	93559.126
Metric	1814.645	986	<0.001	0.963	−0.002	0.039 [0.036,0.041]	0.001	0.045	93340.381
Scalar	1868.589	1,030	<0.001	0.962	−0.001	0.038 [0.035,0.041]	−0.001	0.045	93064.383

### 4.2. Descriptive results

Table 7 shows descriptive results and correlations between research variables. The average mean for each variable ranged from 2.65 to 3.75. Adaptive teaching and output = input achieved the same (and highest) score. A significant correlation existed between the flexible grouping of Chinese lower secondary school teachers alongside output = input and adaptive teaching, accompanied by the growth mindset of teachers. A moderate correlation was observed between flexible grouping and output = input; otherwise, low correlation values were observed.

**Table 7:** Descriptive statistics and Pearson correlation coefficients between DI variables.

**Variables**	***M***	**SD**	**1**	**2**	**3**	**4**	**5**
**1. Adaptive teaching**	3.75	0.63	1.00				
**2. Growth mindset**	3.39	0.86	0.334***	1.00			
**3. Ethical compass**	2.65	0.84	−0.071*	0.053	1.00		
**4. Flexible grouping**	3.66	0.65	0.702***	0.397***	0.051	1.00	
**5. Output = input**	3.75	0.65	0.609***	0.224***	0.151***	0.431***	1.00

***p < 0.001, *p < 0.05.

### 4.3. Predictors of differentiated instruction practice (i.e., adaptive teaching)

**Figure 3:**
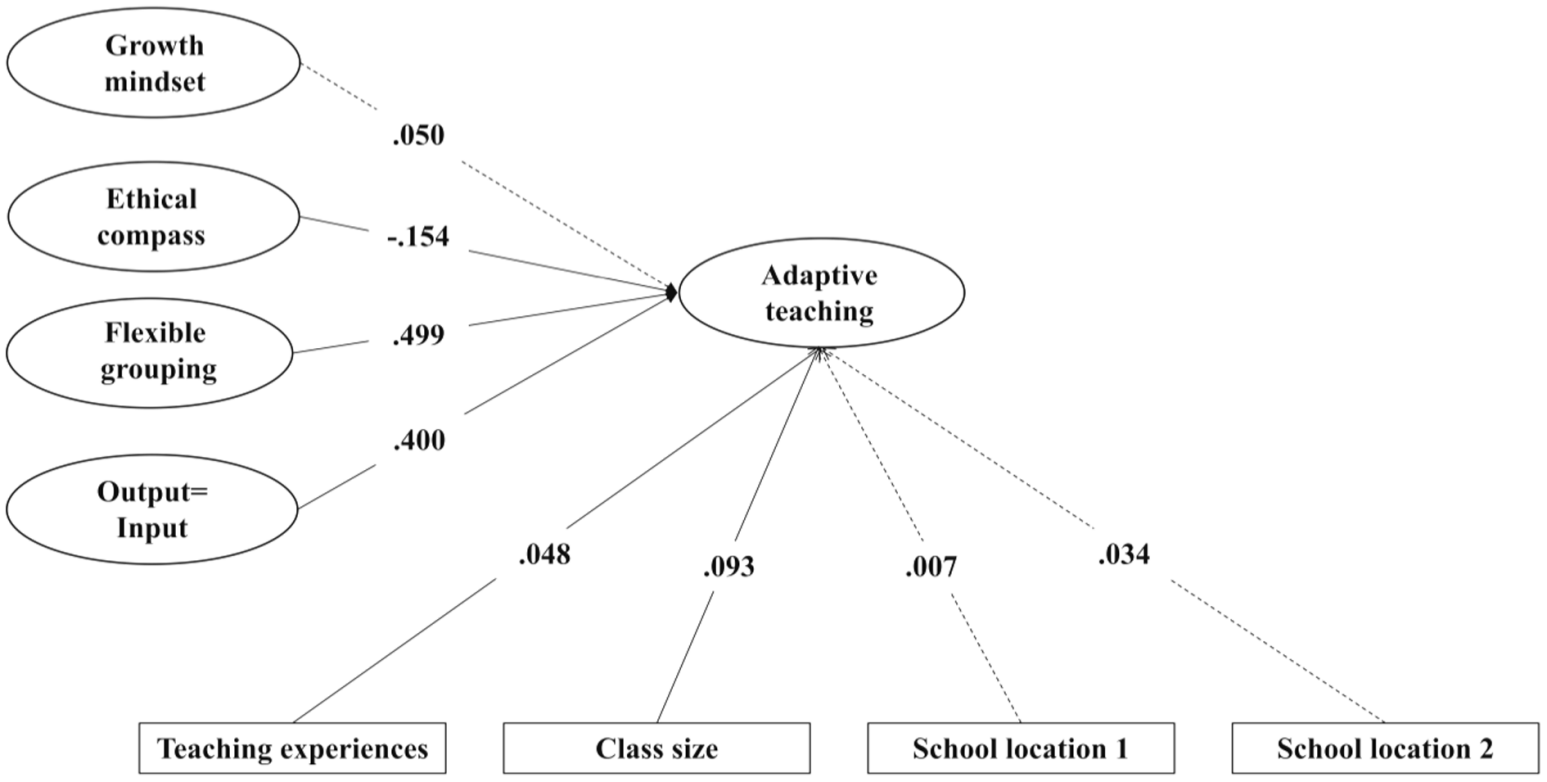
Structural equation modeling of the conceptual model; *N* = 1,689. School location 1: Town (Base = rural schools); School location 2: City (Base = rural). Standardized path estimates are reported. The path estimates in solid line that were significant while estimates in dashed line were not significant.

Structural modeling results indicated that an acceptable conceptual model (SBχ^2^ = 1132.465, df = 412, SBχ^2^/df = 2.749, CFI = 0.970, TLI = 0.966, SRMR = 0.050, RMSEA = 0.032). Flexible grouping, output = input, ethical compass, teaching experience, and class size largely explained teachers’ DI practice (*R*^2^ = 62.3%; Figure 3). Table 8 shows that the effects of output = input, flexible grouping, teaching experience, and class size on teachers’ DI practice were statistically significant, and these variables served a beneficial function in the model. However, the results of the impact of growth mindset and school location on DI practice were not significant; ethical compass was observed to have a negative impact on DI practice.

**Table 8:** Direct effects of teachers’ growth mindset, ethical compass, flexible grouping, output = input, teaching experience, class size, and school location on DI practice (i.e., adaptive teaching).

**Standardized effects**	**β**	**SE**	***t***
Growth mindset → DI practice	0.05	0.029	1.738
Ethical compass → DI practice	−0.154	0.024	−6.305***
Flexible grouping → DI practice	0.499	0.029	17.068***
Output = input → DI practice	0.400	0.026	15.161***
Teaching experience → DI practice	0.048	0.019	2.574**
Class size → DI practice	0.093	0.017	5.353***
Town (Base = rural) → DI practice	0.007	0.02	0.333
City (Base = rural) → DI practice	0.034	0.021	1.61

“Town” and “city” as dummy variables.

*R*^2^ = 0.623; ***p < 0.001; **p < 0.01.

## 5. Discussion

As the first study to explore teachers’ DI practice in Chinese mainland schools, this study extends previous work, which has lacked understanding of Chinese teachers’ perceptions and implementation of DI. Most prior research has not developed and validated DI-related instruments in non-Western counties, and although the DI-Quest had been studied in Hong Kong schools (Yuen et al., 2022), a replication study in the context of Chinese mainland schools was needed, since the school cultures and educational systems of Hong Kong and China differ (Malinen et al., 2012). Therefore, the current study makes up for this deficiency and fills a gap in the literature by adding the experiences of Chinese mainland schoolteachers to the extant literature on DI.

### 5.1. Factor structures in the Chinese mainland version of the DI-Quest

This study is the first examine the psychometric properties of the DI-Quest instrument in the context of Chinese mainland lower secondary schools. The CN-DI-Quest verified the same five factors as the original and Hong Kong versions (Coubergs et al., 2017; Yuen et al., 2022). This study omitted four items, which contrasts with Yuen et al. (2022), who removed 12. Reviewing the items eliminated from the original instrument helped us, in some cases, to better understand DI in Chinese mainland schools. For example, EC2 (*“The curriculum is overloaded on content and goals”*) and AP4 (*“Every student will receive the same assessment”*); these two items may have suggested that Chinese teachers are curriculum-oriented and teacher-centered, but DI, according to the theory, should be oriented to students (Tomlinson and Imbeau, 2010). Regarding the other two deleted items, FG4 (*“During my lessons*, *students need to work together in order to progress in their learning process”*) and FG8 (*“I differentiate by switching between working with heterogeneous and homogeneous groups”*) implied that Chinese teachers may not consider flexible grouping strategy during their DI implementation. This may be explained by the class sizes in Chinese mainland schools. As Table 2 shows, 75.3% of classes in this study contained more than 40 students. So many students in one classroom make it difficult for teachers to group them flexibly, due to the increased diversity, number of groups formed, challenges to classroom management, and time taken for interaction between students and the teacher (e.g., Suprayogi et al., 2017; Aldossari, 2018). Another plausible explanation is that Chinese mainland teachers are discouraged from grouping students flexibly, which is supported by Gaitas and Martins (2017) who reported that teachers encounter barriers to the adjustment of teaching procedure and classroom management when grouping students during teaching.

### 5.2. Predictors of teachers’ differentiated instruction practice

This study has also shown that Chinese mainland teachers’ DI philosophies, principles, teaching experience, and class size have a significant impact on their self-reported DI practice (adaptive teaching). We achieved this by connecting the DI-Quest instrument with teacher-levels and context-level variables. Our findings indicate that flexible grouping is an essential predictor of teachers’ DI practice in the context of schools in China, which is not surprising, since other studies have found that teachers who prefer to flexibly group students in heterogeneous and homogeneous combinations tend to use DI more often (Tomlinson et al., 2003; Ford, 2005; Coubergs et al., 2017). To adopt DI practice, teachers must become skilled in switching groups in various ways; this corresponds with the research findings whereby the integration of diverse forms of flexible grouping strategy helps students to achieve learning outcomes at appropriate levels (Whitburn, 2001; Castle et al., 2005).

The results of this study have also reported that the second predictor of the practice of DI is output = input; this indicates that teachers are more likely to use differentiation techniques in their practice if they consider the feedback from, and evaluation of, students as teaching resources on which to base their next lesson plans. This study’s findings are consistent with those of Coubergs et al. (2017) and Griful-Freixenet et al. (2021), both of whom identified output = input as a strong variable to explain DI practice. The logic behind the DI theoretical framework also explains this positive outcome, in which teachers are assisted by continuous assessment at every stage of instruction to adapt both teaching and learning plans to the needs of students (Hall, 2002; Tomlinson and Moon, 2014).

This study indicated that teaching experience was found to be the third predictor of DI practice. The experienced teachers, namely those with more than 5 years of teaching behind them, who took part in this research have higher levels of DI philosophy and practice. This contradicts the findings of McMillan (2011), but corroborates Garrett (2017) and Suprayogi et al. (2017) assertion that novice teachers with less than 5 years’ experience were associated with a lower frequency of DI practice. This may be explained by the professional development and training of teachers (VanTassel-Baska et al., 2008; Suprayogi et al., 2017), whereby only after years of training and experience can teachers integrate DI-related content and knowledge to move from fact-based programs to authentic investigations and become an educational subject matter expert (Moosa and Shareefa, 2019). Novice teachers are trained in DI during initial teacher training, since at that stage most lack insight concerning relevant variations among students or cannot identify differentiation needs (Dack and Triplett, 2020). Class size was also reported to predict DI practice significantly in this study. This confirmed the findings of Tomlinson et al. (2003) and Suprayogi et al. (2017), in which DI practice is acutely required, to accommodate students’ differences in larger classes, possibly because an increase in student numbers increases the extent diversity in students and learning needs (Dixon et al., 2014), which requires teachers to adopt more differentiated approaches to addressing such large-scale learning diversity (Smale-Jacobse et al., 2019).

Another predictor in this study was teachers’ ethical compass, which had a negative impact on DI practice. This resonates with the findings of Coubergs et al. (2017) and Griful-Freixenet et al. (2021), who have stated that teachers who focus on students’ learning to guide their teaching practices rather than use unquestioning compliance with the curriculum as a teaching guide, seemed to use more differentiation in their practice. These findings echo Tomlinson (2014), wherein DI is integrated with a high-quality curriculum in accordance with students’ needs. Therefore, if teachers focus primarily on external variables such as discipline or curriculum structure, they tend to adopt traditional practices without considering students’ needs (Nowell, 1992; Coubergs et al., 2017).

## 6. Limitations and recommendations for future research

While this study is a pioneering work that explores the impact of teachers’ DI philosophies and principles on their DI practice in the Chinese mainland, it has been subject to certain limitations that could be mitigated in future research.

Firstly, the DI-Quest instrument in this study was a self-reporting survey, which may have prompted some teachers to give socially desirable, rather than completely accurate, responses. The use of other research methods, such as classroom observation, videos, and individual and group interviews, may overcome this shortcoming. Also, Graham et al. (2021) argued that more DI research should be conducted in diverse countries around the world. Consequently, replication studies using the DI-Quest should be conducted in different school areas and contexts.

The present study uses a cross-sectional design, which limits the capacity to demonstrate causal interpretations; we recommend that longitudinal and experimental research studies should be conducted, to reveal more about DI in Chinese mainland schools. Furthermore, this study has surveyed only teachers working in central and western China; regional differences are among the variables that generate disparity in Chinese education (Yang et al., 2014), and further research could invite respondents from eastern China, and assess whether outcomes are similar there.

Additionally, this study has focused on teachers’ perceptions of their DI philosophies and practices according to the DI-Quest model; however, in the classroom setting, students are critical. Future research might helpfully explore is how students perceive the differentiated teaching practices deployed by their teachers since consideration of students’ experiences in the course of DI practice helps teachers to hone their approaches (Pozas et al., 2021).

Finally, previous studies have reported that other variables, like teachers’ self-efficacy and attitudes toward DI, are predictors of DI (e.g., Coubergs et al., 2017; Letzel et al., 2020). Hence, future empirical studies should connect more extensive quantitative instrumentation, including measures of teachers’ self-efficacy and attitudes toward DI, with the DI-Quest instrument, together to understand teachers’ DI practice.

## 7. Conclusions and implications

This study provides a novel, valid and reliable instrument for future research of DI in mainland Chinese contexts. In this regard, the findings of this study have confirmed the importance and significance of the DI-Quest in non-Western countries, which will support further comparative studies between mainland China and Western countries. The current study also offers implications for educational officers to concentrate on the professional advancement of teachers with regard to DI, as (1) teachers’ flexible grouping, output = input, and teaching experiences influence their DI practice positively and significantly; (2) the role of ethical compass in teachers’ DI practice is negative. Thus, a feasible approach to actualizing DI is through further assistance, such as professional advancement. Specifically, teachers are expected to undertake training programs and learn how to organize various forms of grouping and evaluation strategies to enhance their DI practice and meet students’ learning needs. To gain proficiency in such skills, teachers must learn when and where to offer differentiated instructions and feedback (Lambert, 1999; Lawrence-Brown, 2004). According to Hall (2002), assessment plays an essential role in DI, and it requires teachers to have a deep understanding of their students; that understanding also functions as the starting point for the diagnosis of students’ differences in readiness, interest, and learning styles. Therefore, through a measure of pre-assessment, teachers can adapt their teaching to respond to students’ learning status and assist students accordingly (Tomlinson, 2001; Hall, 2002).

Moreover, to ensure that teachers’ perceptions in terms of ethical compass are oriented to students, rather than the curriculum, it is essential that teachers partake in regular discussions and collaborations about learners’ differences, curriculum adaptation, teaching objectives, and/or subject knowledge (Tomlinson et al., 2003; Woolfolk, 2010). Following exchanges of experience, teachers may adjust their curriculum-oriented beliefs and accept the varied characteristics of their students, thereby connecting their perceived insights to classroom reality (Eisenberger and Shanock, 2003; Hattie, 2005). In this regard, school leaders should support and encourage teachers to adopt DI by cultivating an inclusive learning atmosphere. Furthermore, the significance of developing professional skills for teachers in accordance with actual teaching experience is reiterated by Bandura (1977). Teachers who have personally experienced the advantages and accomplishments of DI can put that experience to good use; it is extremely significant to consider reflections on previous teaching experiences, as well as interaction with peers (Wertheim and Leyser, 2002; He and Levin, 2008).

To conclude, this study has validated the DI-Quest instrument in a mainland Chinese school context and has explored the extent of teachers’ DI practice by addressing their DI philosophies and principles. The CN-DI-Quest appears to be a promising instrument for future research, and retains the five-factor structure of the original and Hong Kong versions, with good reliability and validity. Chinese educators may consider how best to use this instrument to understand teachers’ perceptions of DI and to improve their DI skills through school-based DI professional development programs. Furthermore, the research method in this study (structural equation modeling) can be implemented in various school contexts, to explore the influence of teachers’ philosophies, principles, teaching experiences, class size, and school location on their DI practice.

## Data availability statement

The raw data supporting the conclusions of this article will be made available by the authors, without undue reservation.

## Author contributions

MB conceived the idea of this study, collected data online, processed data by using SPSS and MPLUS, and wrote the full manuscript. KS was a promoter who was responsible for mentoring and reviewing the whole process of writing this article. CZ worked as the co-promoter for editing the original draft. All authors provided the critical feedback and approved the final manuscript.

## Conflict of interest

The authors declare that the research was conducted in the absence of any commercial or financial relationships that could be construed as a potential conflict of interest.

## Publisher’s note

All claims expressed in this article are solely those of the authors and do not necessarily represent those of their affiliated organizations, or those of the publisher, the editors and the reviewers. Any product that may be evaluated in this article, or claim that may be made by its manufacturer, is not guaranteed or endorsed by the publisher.
